# Parasite Removal Improves Reproductive Success of Female North American Red Squirrels (*Tamiasciurus hudsonicus*)

**DOI:** 10.1371/journal.pone.0055779

**Published:** 2013-02-08

**Authors:** Jesse E. H. Patterson, Peter Neuhaus, Susan J. Kutz, Kathreen E. Ruckstuhl

**Affiliations:** 1 Department of Biological Sciences, University of Calgary, Calgary, Alberta, Canada; 2 Faculty of Veterinary Medicine, University of Calgary, Calgary, Alberta, Canada; University of Manitoba, Canada

## Abstract

In order to evaluate potential reproductive costs associated with parasitism, we experimentally removed ectoparasites from reproductive female North American red squirrels (*Tamiasciurus hudsonicus*). Body mass and overwinter survival of mothers, days to juvenile emergence, juvenile survival from birth to emergence, and body mass of juveniles at emergence were all compared to those of untreated (control) animals. Ectoparasite removal did not affect the body mass of mothers throughout the lactation period and overwinter survival of mothers did not differ between treatments and controls. Likewise, there was no effect of treatment on the number of days to juvenile emergence. However, treated mothers raised offspring that were significantly heavier (11%) than controls at emergence. Juveniles from treated mothers were also 24% more likely to survive from birth to emergence. Our results indicate that ectoparasites impose costs on the reproductive success of female red squirrels and that ectoparasites have the potential to influence red squirrel life-histories and population dynamics.

## Introduction

Two of the central tenets of life-history theory are that natural selection maximizes fitness and that fitness-related traits are constrained by trade-offs given limited resources [1]. By imposing considerable energetic costs on their hosts [2]–[4], parasites have been implicated in the development, expression and evolution of many life-history traits [5], [6]. Of particular interest to evolutionary ecologists is the role that parasites play in shaping the fitness of their hosts. In mammals, an increase in maternal investment can increase offspring condition and/or mass, which in turn may improve offspring fitness [7]. Parasites impose considerable energetic and behavioural costs on their hosts [3], [8]–[10]. Hosts therefore have to deal with trade-offs between current reproduction, future reproduction, maintenance, survival and the costs associated with parasites. To address these complex trade-offs, parents may adjust the number and quality of their offspring in order to optimize their own reproductive success.

Experimental tests of the relationship between host reproductive success and parasitism have been scant, especially in mammals [11], [12]. Neuhaus [11] and Hillegass et al. [12] both utilized anti-parasitic insecticides to experimentally remove ectoparasites from female ground squirrels (*Urocitellus columbianus* and *Xerus inauris*, respectively). These experimental removals resulted in significant increases in the reproductive success of the studied females [11], [12]. Correlative tests have been more ambiguous in mammals. For instance, ectoparasite intensity was positively correlated with the reproductive success of adult male North American red squirrels (*Tamiasciurus hudsonicus*) [13], while ectoparasite intensity did not correlate with the reproductive success of muskrats (*Ondatra zibethicus*) [14]. However, greater ectoparasite intensity has been correlated with reduced overwinter survival, and thus recruitment, of juvenile alpine marmots (*Marmota marmota*) [15]. Comparatively more work has been done with ectoparasite interactions in birds; nonetheless, the experimental and correlative results with respect to host reproductive success appear similarly equivocal [16]–[25]. The effects of ectoparasites on host reproductive success and physiology are variable and dependent on environmental conditions, host susceptibility, host life-history, and host-parasite coevolutionary ties.

To evaluate the impact of ectoparasites on the reproductive success and survival of North American red squirrels, we experimentally removed ectoparasites from reproductive females shortly after mating. We compared juvenile mass, juvenile survival from birth to emergence, and juvenile emergence dates between control and treatment litters, as well as the body mass and overwinter survival of parasitized and non-parasitized mothers. Since ectoparasites can reduce the amount of energy available to support lactation, body maintenance, offspring growth, and survival [26], we hypothesized that ectoparasite removal would result in heavier mothers, increased overwinter survival of mothers, heavier juveniles at emergence, and a higher percentage of juvenile survival between two important life-history stages (birth and emergence). If parasite removal resulted in faster-growing juveniles, we hypothesized that these juveniles, while not necessarily heavier than control juveniles, would experience advanced emergence dates compared to controls. Earlier emergence dates can convey important advantages to juvenile red squirrels, such as increased overwinter survival [27]. Measuring the effects of ectoparasites on female reproductive success, offspring mass and offspring survival will contribute to our understanding of the coevolution of host and parasite life-history traits.

## Materials and Methods

### Study Area

We conducted this study in Sheep River Provincial Park, Alberta, Canada (110° W, 50° N; 1500 m) in the foothills of the Rocky Mountains between 2010 and 2011. Study sites were composed of mature second-growth subalpine forest (∼80–100 years old) dominated by white (*Picea glauca*) and black (*P. mariana*) spruce, and interspersed with aspen (*Populus tremuloides*), balsam fir (*Abies balsamea*) and lodgepole pine (*Pinus contorta var. latifolia*).

### Study Species

Red squirrels are small (<250 g) rodents common in coniferous habitat throughout much of North America. They defend food-based territories containing at least one central food cache (midden) year-round [28]. Red squirrels are promiscuous and they exhibit a scramble-competition mating system wherein a female mates with an average of 7 males in a single day [29]. Females are in estrus for one day and males congregate on her territory to compete for access [30]. Reproductive females generally produce a single litter each year following a mean gestation period of ∼33 days [30]. Juveniles emerge from the nest approximately 40 days after birth and are fully weaned and independent at about 70 days following birth [30]. Mean litter sizes range from 3.2 to 5.4 depending on geographic location and food availability [30], [31]. This species displays a small degree of sexual dimorphism, with adult males being slightly (5–10%) heavier than females [32].

Red squirrels in the Sheep River population are known to host at least three species of fleas (*Orchopeas caedens*, *Monopsyllus vison, Taropsylla coloradensis*), two species of lice (*Hoplopleura sciuricola, Neohaematopinus sciurinus*), and one species of tick (*Ixodes angustus*) [33].

### Animal Capture and Experimental Design

We captured squirrels from late April to late September in 2010 and 2011 at three ∼6.75 ha sites by use of live-traps (H. B. Sherman Traps, Inc., Tallahassee, Florida) baited with peanut butter. One grid (10×4 trap pattern) was established at each site with forty traps spaced 50 m apart. Fleas were counted, but never removed, from all captured animals used in the study by systematic searching and combing with a fine metal flea comb. We searched the entire body of each squirrel for two-minutes: dorsally from the tip of the tail to the ears and ventrally from the tip of the tail to the forelimb in 1 cm swatches by combing and blowing on the fur to expose the skin while counting any observed fleas. We paid particular attention to the ears, groin/genitals and underarms, as fleas appeared to favour these areas. Any fleas found in the handling bag following the release of the animal were also included in the prevalence and intensity estimates. Prevalence (number of infected individuals in a sample of hosts [34]) and intensity (number of fleas per infected individual [34]) were determined for all mothers and their offspring. We did not quantify prevalence or intensity of lice, mites or ticks.

We captured adult females, weighed them to the nearest gram using a spring scale (Pesola AG, Baar, Switzerland), attached unique ear tags (Monel #1, National Band and Tag Co., Newport, Kentucky) with unique combinations of coloured washers (National Band and Tag Co., Newport, Kentucky) and radio-collared them (SOM-2190, Wildlife Materials, Inc., Murphysboro, Illinois). In 2010 and 2011, we randomly assigned mothers to either the treatment or control group, initially based on the flip of a coin and alternating thereafter. We treated all females in the treatment group once every thirty days with 0.15 ml/kg of K9 Advantix (8.8% imidacloprid, 44.0% permethrin; Bayer HealthCare LLC, Animal Care Division, Shawnee Mission, Kansas) applied to the skin between the shoulder blades. This drug combination is highly effective at killing ectoparasites (including fleas, lice, mites and ticks) and has low toxicity in mammals, including squirrels, at the topical doses used [35], [36]. We initiated the treatment of squirrels 2–3 weeks prior to parturition to ensure that the medication had sufficient time to remove ectoparasites from the mother and from the nest. We handled and trapped the control animals in exactly the same manner and with equal effort as the treatment animals, but did not give them the medication. We conducted all trapping in the morning (7h00–10h00) to ensure consistency in body mass measurements. If individuals were captured on consecutive days, the average mass was used in the analysis.

Shortly after parturition, we located nests using radio-telemetry. We weighed (±0.5 g; Pesola AG, Baar, Switzerland), sexed and aged [32] all offspring. We then gave offspring unique ear notches and reentered the nest when the offspring were ∼25–30 days old to ear-tag them. Several days before juvenile emergence was expected, we tracked mothers to the nest and observed the nest area for any emergent juveniles twice daily until emergence occurred. When juveniles emerged from the nest (mean: 41.5 days after birth, range: 38–44 days after birth) we captured and weighed the mother and her offspring. To control for the mass of individual mothers, we compared mass differences (mass loss or gain) across subsequent life-history events (birth, emergence and weaning). We did not include cases of complete post-parturition litter loss in the analysis.

We determined overwinter survival of mothers by setting traps on and in the vicinity (within 50 m) of her territory and by trapping the same grids used the previous summer between May 1 and June 15, 2011. A trapping pattern of three days open, three days closed was followed (totalling approximately 840 trap-nights per individual). Territory ownership rarely changes across years with the exception being when mothers bequeath their territories to one of their offspring and move to an unoccupied nearby territory or when territory owners die [37]–[39]. As such, trapping and observations of squirrels on and around known territories should give an accurate representation of overwinter survival of adult squirrels.

### Data Analysis

All data were checked for normality and transformed if necessary. Where data could not be normalized, nonparametric tests were used. Proportional data were normalized using an arcsine transformation. All measures of prevalence and median intensity of fleas, as well as their associated 95% confidence intervals (CI), were determined using Quantitative Parasitology version 3.0 [40]. We used Sterne’s exact method to determine the 95% CIs for parasite prevalence [40]. All other statistical analyses were done in R version 2.12 [41]. We tested for year effects with respect to offspring survival, offspring mass at emergence, and emergence dates (number of days from birth) using generalized linear models (GLM) with year, parasite treatment and mother’s mass at emergence as random variables. When no year effects were found, the data were pooled as treatment and control groups in comparative analyses. All comparisons between treatment and control groups were tested using two sample *t* tests, except for comparisons of mother’s mass differences, which were analyzed using nonparametric Mann-Whitney *U* tests. In each year, we followed 12 mothers from parturition through to juvenile emergence and, in each year, 6 mothers were treated and 6 were left as controls. For some of the analyses, we either pooled mothers and litters (n = 24) to control for certain effects of interest (i.e., year) or we pooled, where possible, the control (n = 12) and treatment (n = 12) groups for comparison tests. All means are reported ±1 standard deviation, unless otherwise noted. For non-significant results, we provide 95% CIs for the effect size as a way of describing the range of effect sizes supported by the data [42]. For parametric data these were based on Cohen’s *d* and for nonparametric data these are based on Cliff’s Δ, and were computed using the “orddom” package in R [43].

### Ethics Statement

Capture and handling of animals followed the Guidelines on Care and Use of Wildlife established by the Canadian Council on Animal Care. The University of Calgary Life and Environmental Sciences Animal Care Committee (protocol #AC11-0088) approved the use of all animals and procedures employed in this study. Permits for this work were obtained from the Fish and Wildlife Division of the Ministry of Environment and Sustainable Resource Development, Government of Alberta and the Parks Division of the Ministry of Tourism, Parks and Recreation, Government of Alberta.

## Results

### Flea Prevalence and Intensity

Mothers were only entered into the experiment, as either a control or a treatment animal, if they were found to host at least one flea at the outset. No fleas were observed on treated mothers two weeks following the initial treatment and throughout the study period. Prevalence of fleas on the litters of treated mothers was 0.0% (95% CI: 0.0–24.0%; n = 12 litters) and 100.0% for control mothers (95% CI: 76.0–100.0%; n = 12 litters) at the time of birth. Prevalence of fleas amongst individual offspring from control mothers at birth was 79.6% (95% CI: 66.0–89.0%, n = 49), when data from 2010 and 2011 were pooled. Median intensity of fleas per individual juvenile from control mothers at birth was 5.0 (95% CI: 4.0–7.0; n = 39). The median intensity of fleas in the entire litter at birth from control mothers was 19.5 (95% CI: 10.0–27.0, n = 12 litters), when data from 2010 and 2011 were pooled. Median intensity of fleas per juvenile from control mothers at emergence was 3.0 (95% CI: 1.5–4.5; n = 19), when 2010 and 2011 data were pooled.

### Mother’s Mass and Survival

Control mothers did not lose or gain significantly more mass than treated mothers between measurement periods. Between parturition and emergence, control mothers lost a mean mass of 1.75±10.69 g and treated mothers lost 1.5±24.12 g (Mann-Whitney *U* = 6.0, *p* = 0.629, Δ = 0.25, 95% CI for effect size: −0.66–0.86) in 2010, while in 2011, control mothers gained 2.0±17.3 g and treated mothers gained 10.4±5.4 g (*U* = 3.5, *p* = 0.8, Δ = 0.22, 95% CI for effect size: −0.75–0.89). Between emergence and weaning, control mothers lost 24.0±16.5 g and treated mothers lost 16.8±25.0 g (Mann-Whitney *U* = 10.0, *p* = 0.686, Δ = −0.25, 95% CI for effect size: −0.86–0.65) in 2010, while in 2011, control mothers lost 36.7±17.4 g and treated mothers lost 28.4±10.1 g (*U* = 5.0, *p* = 0.176, Δ = 0.58, 95% CI for effect size: −0.30–0.93). Between parturition and weaning, control mothers lost 25.8±7.9 g and treated mothers lost 18.3±6.2 g (Mann-Whitney *U* = 4.0, *p* = 0.314, Δ = 0.50, 95% CI for effect size: −0.44–0.92) in 2010, while in 2011, control mothers lost 25.8±5.6 g and treated mothers lost 13.4±10.6 g (*U* = 2.0, *p* = 0.229, Δ = 0.67, 95% CI for effect size: −0.39–0.97). Overwinter survival of mothers did not differ amongst treatment and control groups between 2010 and 2011, as two control mothers and two treated mothers from 2010 were not found in 2011.

### Juvenile Mass and Survival

There was no effect of year or mother’s mass at emergence on the mass of juveniles at emergence (*t* = −0.608, *p* = 0.546, n = 24) or on offspring survival from birth to emergence (*t* = 1.959, *p* = 0.074, n = 24) when treatment, mother’s mass and year were jointly entered into a GLM. As such, data were pooled across years. Treated mothers raised offspring that were, on average, 11.6 g (11.1%) heavier at emergence (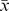 = 116.2±14.6 g, n = 12 mothers) than control juveniles (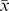 = 104.6±14.2 g, n = 12 mothers; *t = *2.547, *p* = 0.0141 when controlling for mother’s mass, mother’s ID, and year in a GLM). On average, 75.2%±17.5% of juveniles from treated mothers (n = 12) survived from birth to emergence, whereas only 51.3%±22.3% of juveniles from control mothers (n = 12) survived the same interval (*t* = −2.350, df = 14.98, *p = *0.0329).

### Emergence Date

Year (*t* = 0.776, *p* = 0.450), mother’s mass (*t* = −0.148, *p* = 0.884) and parasite treatment (*t* = −0.251, *p* = 0.805) were not significant predictors of juvenile emergence date in a GLM (n = 24 litters). When treated (n = 12) and control (n = 12) mothers were compared, treatment was not found to affect the date of litter emergence (*t* = −0.215, df = 14.65, *p* = 0.833, *d* = −0.01, 95% CI for effect size: −0.52–0.87). Average number of days from birth to emergence for treatment and control litters was 41.4±1.9 days and 41.6±1.1 days, respectively.

## Discussion

Our study shows that the removal of ectoparasites can directly affect host reproductive success. Our results suggest that ectoparasites have a substantial and persistent negative effect on the reproductive success of red squirrel mothers, in that we detected costs to reproductive output due to parasitism across years and despite a small sample size. Not only did our broad ectoparasite removal affect the mass of juveniles at emergence, but it also enhanced survival probabilities of juveniles between successive life-history stages (birth and emergence). Similarly, access to food resources has been found to influence the survival of juvenile red squirrels from birth to emergence, although food supplementation only affected juvenile survival and did not improve the body mass of juvenile red squirrels at emergence [27]. However, much like access to food, parasites factor into the energetic equation of their hosts. Parasitised offspring, by losing energy directly to parasites [10], likely have less energy to invest into their own growth and condition regardless of the mother’s investment. While we only quantified fleas in this study, we do not attribute our findings solely to fleas since lice, mites and ticks also parasitize red squirrels, and all of these ectoparasites would have been removed from treated mothers through our anti-parasite treatment.

Our results are consistent with previous findings, which show that parents are able to produce more, higher-quality offspring when ectoparasites are removed [11], [12], [44]. For instance, removal of ectoparasites from female Columbian ground squirrels (*Urocitellus columbiensis*) resulted in an increase in total litter mass at weaning and number of emergent juveniles [11]. Similarly, Cape ground squirrel (*Xerus inauris*) mothers with experimentally reduced parasite intensities raised more offspring than controls [12]. Comparable findings have also been reported for birds. In barn swallows (*Hirundo rustica*), cliff swallows (*Hirundo pyrrhonota*) and great tits (*Parus major*), experimental treatment of nests against ectoparasites resulted in improved survivorship of nestlings between hatching and fledging, as well as heavier chicks at fledging [22], [44], [45]. Juvenile barn swallows had longer nestling periods than did controls when ectoparasites were experimentally removed from the nest [22]. Parasitized nestlings may have fledged earlier as a form of parasite escape, despite facing reduced survival probabilities as a result [22]. We did not find any effect of parasite treatment on the number of days to emergence for red squirrel juveniles, contrary to our prediction that non-parasitized juveniles might emerge earlier if their growth-rates were accelerated. For red squirrels, early emergence conveys advantages such as greater access to available territories, a longer period of time in which to acquire and store food resources, and a higher likelihood of overwinter survival [27]. Despite the advantages of earlier emergence, the actual number of days between birth and emergence does not appear to be very flexible. Instead, mothers in good condition may mate earlier in the season, thereby ensuring their young benefit from early emergence [27]. Our experimental design did not manipulate the date of mating as all females were randomly entered into the experiment after mating had occurred.

In highly variable environments, it may be beneficial for females of short-lived iteroparous species, such as red squirrels (female red squirrels surviving to 1 year of age have an average lifespan of 3.5 years [31]), to invest heavily in current reproduction if reproductive conditions (i.e., food availability, population density, parasitism) are favorable, even if it does trade off with future reproduction or survival [46]. However, we did not find any effect of parasitism on the overwinter survival of mothers, which is consistent with other red squirrel populations [47]. In red squirrels, reproductive success appears to vary in response to prevailing environmental conditions, such as food availability [48] and, as shown here, parasitism. Our findings suggest that female red squirrels maintain an optimal body mass regardless of their parasite infection or reproductive output, possibly to optimize their future survival and reproductive potential. If they lack the energy required for bodily maintenance and reproduction, females appear to invest less into their current offspring. This would explain the impact of the experimental parasite removal on juvenile size and survival, and the lack of impact on mother’s mass and survival. Unlike other squirrel species, red squirrels store much of their energy in the form of conifer cones in a midden rather than as body fat. Thus, it is not surprising that we could not detect an effect of ectoparasites on female body weight, although the same trend was also observed in Columbian ground squirrels, who do store their energy as fat [11]. There may also have been effects on hoard depletion, which we did not measure. While our non-significant findings are biologically relevant and supported by findings in other red squirrel populations, readers should interpret these results with caution. Due to small sample sizes, our power to detect an effect where one may have been present was low, especially for mother’s mass differences, as shown by the effect size confidence intervals [42]. The hypotheses tested here for which significant differences between treatment and control groups were not found may in fact be Type II error. However, the effect size and corresponding confidence interval for days to emergence are consistent with the null hypothesis of no effect of parasitism and, therefore, our conclusion is supported by our data. Future research on the effects of ectoparasitism on adult survival, midden depletion, and juvenile emergence dates, as well as on the effects of endoparasites on various life-history components of red squirrels, is warranted.

Many populations and species may be faced with increased parasite infection risk due to range expansions of pathogens and hosts, changes in parasite communities, and anthropogenic environmental change [49], [50]. Ecologists rarely consider the effects of parasites when predicting population demographics and informing management practices; however, parasites have important implications for individual host reproductive success, host life-histories, and host population viability [11]–[13], [51], [52]. Our study is a rare investigation of the consequences of parasitism on observed host life-history traits in a natural population of free-ranging mammals and is, to our knowledge, the first study to directly assess the effects of ectoparasites on the survival of mammalian offspring from birth through to emergence.
